# Comparison of rapid diagnostic test Plasmotec Malaria-3, microscopy, and quantitative real-time PCR for diagnoses of *Plasmodium falciparum* and *Plasmodium vivax* infections in Mimika Regency, Papua, Indonesia

**DOI:** 10.1186/s12936-015-0615-5

**Published:** 2015-03-05

**Authors:** Liony Fransisca, Josef Hari Kusnanto, Tri Baskoro T Satoto, Boni Sebayang, ᅟ Supriyanto, Eko Andriyan, Michael J Bangs

**Affiliations:** Public Health & Malaria Control, International SOS, PT. Freeport Indonesia, Kuala Kencana, Papua Indonesia; Center for Tropical Medicine, Faculty of Medicine, Gadjah Mada University, Yogyakarta, Indonesia; Public Health Department, Faculty of Medicine, Gadjah Mada University, Yogyakarta, Indonesia

**Keywords:** Malaria, Rapid detection test, Microscopy, PCR, Sensitivity, Specificity

## Abstract

**Background:**

The World Health Organization recommends malaria be diagnosed by standard microscopy or rapid diagnostic test (RDT) before treatment. RDTs have been used with greater frequency in the absence of matching blood slide confirmation in the majority of RDT reported cases in Mimika Regency, Papua Province, Indonesia. Given the importance of RDT in current health system as point-of-care tool, careful validation of RDT product performance for providing accurate malaria diagnosis is critical.

**Methods:**

Plasmotec Malaria-3 (XW-P07) performance was evaluated by comparing it with paired blood film microscopy and quantitative real-time PCR (qPCR). Consecutive whole blood samples were derived from one clinic in Mimika as part of routine passive malaria case detection. RDT results were read by two trained technicians and interpreted by consensus. Expert microscopic examination of blood slides was cross-checked by observer-blinded second reader and a third examiner if discordant between examinations. qPCR was used as the ‘gold standard’, followed by microscopy for the outcome/disease variable. Comparison analysis included sensitivity (Sn), specificity (Sp), positive and negative predictive values (PPV & NPV), and other diagnostic screening performance measures for detecting *Plasmodium falciparum* and *Plasmodium vivax* infections.

**Results:**

Overall malaria positive samples from qPCR was 42.2% (175/415 samples); and from matching blood slides 40.5% (168/415) of which those infections with relatively low parasite densities ≤100/μl blood was 5.7% of *P. falciparum* and 16.5% of *P. vivax* samples examined. Overall RDT performance when compared with microscopy for detecting *P. falciparum* was Sn:92%, Sp:96.6%, PPV:88%, NPV:97.8%, Kappa:0.87; and for *P. vivax* Sn:72.9%, Sp:99.1%, PPV:95.4%, NPV:93.4%, Kappa:0.79. Overall RDT performance when compared with qPCR for detecting *P. falciparum* was Sn:92%, Sp:96.6%, PPV:88%, NPV:97.8%, Kappa:0.87; and for *P. vivax* Sn:66%, Sp:99.1%, PPV:95.4%, NPV:90.9%, Kappa:0.73.

**Conclusions:**

Plasmotec Malaria-3 test showed good overall performance scores in precision for detecting *P. falciparum*, but lower values regarding sensitivity and negative likelihood ratio for detecting *P. vivax*, a finding partly associated with greater frequency of lower density *P. vivax* infections compared to *P. falciparum* in this study. In particular, the negative likelihood ratio (>0.1) for *P. vivax* detection indicates RDT lacked sufficient discriminating exclusion power falling below general acceptance criteria.

## Background

Malaria remains a serious public health problem in Indonesia. Approximately 45% of the populations across the archipelago are at risk for malaria infection [1], with 417,819 confirmed malaria cases in 2012. However, the national annual parasite incidence (API) i.e., the number of people per 1,000 populations that experienced at least one case of malaria in a 12-month period, had decreased from 4.68‰ in 1990 to 1.69‰ in 2012. This is an encouraging development towards an overall national target API of below 1‰ by year 2030 [2].

While several areas have witnessed significant reductions in malaria prevalence, other regions (e.g., eastern Indonesia) have remained problematic for performing effective control strategies because of remoteness, lack of adequate resources and sufficient budgets to combat both vectors and parasites. Papua and West Papua provinces of Indonesia, located on the western half of the island of New Guinea, have the highest malaria burdens in the country, with recent province-wide APIs of 60.6‰ and 52.3‰, respectively [3]. All four species of *Plasmodium* parasites are present in Papua, with *Plasmodium falciparum* and *Plasmodium vivax* as the most common infections, followed with far less frequency by *Plasmodium ovale* and *Plasmodium malariae*. In high transmission areas, mixed species infections are not uncommon. Mimika Regency, covering a vast area of the southern part of Papua Province, had an API of 531.3‰ in 2012 with an overall *P. falciparum*/*P. vivax* case infection ratio of 1.3:1 [4]. Other report had estimated the average API closer to 876‰ in the immediate Timika area, the capital of Mimika, and where the vast majority of the population resides [5].

The World Health Organization (WHO) recommends all clinically suspected malaria cases have parasitological confirmed diagnosis, using either a malaria-specific rapid diagnostic test (RDT) or direct visualization of parasites using microscopy, before treatment [6]. For more than a century, use of microscopy has been considered the ‘gold standard’ for malaria diagnosis, species identification, and to quantify parasitaemia [7]. Various public and private health care facilities in the Timika area can perform standard microscopic diagnosis of malaria, but this is often compromised by the poor condition and maintenance of the microscope and the irregular availability of a trained laboratory technician. In many of the remote villages in the Mimika Regency (particularly those without electricity, skilled staff, or microscopist) and most public-run clinics, only RDT is used for malaria diagnosis. INDEC Diagnostics (Jakarta, Indonesia) manufactures a multi-panel malaria RDT Plasmotec Malaria-3 (hereafter referred to using the product catalog number XW-P07) that meets ISO 13485:2003 standards [8]. Including the company’s internal assessment of the RDT, there is only one known published evaluation of this product [9] that occurred in southern Sumatra; therefore, a performance evaluation of this RDT was deemed prudent if this product was to be recommended for wider use in the Mimika area. RDT quality (accuracy and precision) is especially important given the infrequent use or absence of routine microscopy or matching blood film confirmation in the majority of instances in Papua.

Among other criteria, a useful and effective RDT must have sufficiently high sensitivity to be able to accurately identify as many ‘true’ malaria cases as possible, especially in areas where reliable microscopy is not available or used infrequently. This is particularly important so that infections can be effectively and specifically treated based on parasite species. The screening sensitivity of an RDT can be influenced by the epidemiological characteristics and infection dynamics in the target population. As parasite antigen concentrations in the blood and parasitaemia levels can vary due to multiple host and parasite factors, the performance level of an RDT can be similarly affected depending on the malaria-endemic population involved in the product assessment [10].

Establishing test performance accuracy for disease screening should be considered before investing and committing to a specific product. Although certain tests may be relatively inexpensive and easy to use, they must be valid and provide consistent reproducible results. Test accuracy describes the diagnostic strength of the association between the predictor variable (RDT result) and outcome variable (disease) as measured against a ‘gold standard’ test. The most common and useful complementary measures for evaluating a test are sensitivity (the proportion of true diseased persons in a population who are test positive – the true positive rate), specificity (the proportion of truly non-diseased persons who are so identified by the test – the true negative rate), and the positive and negative predictive values (the probability that the disease is present or not when the test is positive or negative, respectively). However, unlike the previous measures, another set of complementary statistics, the positive and negative likelihood ratios, are less likely to be affected by background disease prevalence [11], and are thus considered among the best measures of test accuracy [12]. Diagnostic likelihood ratio represents the odds ratio that a positive (or negative) test will be observed in an infected population compared to the odds that the same result will be observed in a non-infected population. A positive likelihood ratio >10 and a negative likelihood ratio <0.1 generally indicate a test holds sufficient merit as a useful diagnostic tool [13]. Lastly, test consistency describes diagnostic reliability (precision) and includes a measure of agreement (Kappa statistic) between one test and another (e.g., new test versus a ‘standard’) using dichotomous variables (malaria positive or negative). A Kappa score of 1 indicates perfect agreement, while 0 indicates the equivalent of chance having produced the apparent agreement [14,15].

The XW-P07 has not been listed, or data presented, as formally evaluated by the World Health Organization’s Malaria RDT Product Testing Program in Rounds 1 through 5 (2008–2013) [10,16]; therefore, the objective of this study was to evaluate the performance of this device as a point-of-care diagnostic test, when compared with expert microscopy and quantitative real-time PCR (qPCR).

## Methods

### Site and procedures

Data collection was conducted in Kuala Kencana, a township near Timika, in April and May 2014. The health facility was selected as the XW-P07 was in current use for routine malaria diagnosis with microscopy. The annual slide positive rate (SPR) at the Kuala Kencana Clinic for 2012 and 2013 was 40% (*P. falciparum*/*P. vivax* ratio = 1.3:1) and 38% (*P. falciparum*/*P. vivax* = 1:1.2) respectively, indicating no significant changes in SPR while having a notable shift between the two years in the proportion of parasite species in favor of *P. vivax*. The clinic is equipped with a modern laboratory and full-time expert laboratory staff and malaria microscopists with access to detailed medical records on all malaria cases reported. The inclusion criteria for study samples included one or more of the following: 1) passive patient visit to clinic through either the emergency or outpatient department; 2) patient presenting with suspected malaria infection that included one or more of these following symptoms: fever, chills, headache, nausea, vomiting, abdominal pain and/or diarrhea, myalgia; 3) patient having lived in a malaria-endemic area or visiting one in the past four weeks; and 4) the patient may have experienced malaria before [17].

Following informed consent by patient adults or minors (with parental/legal guardian’s consent), using sterile procedures, a single 3 ml venous blood sample was collected in a glass Vacutainer® tube containing 5.4 mg dipotassium (K_2_) EDTA (Becton, Dickinson and Co., New Jersey, USA) from each suspected malaria case. Blood was used to prepare an RDT with a matched thick and thin blood slide for microscopy, and qPCR testing. Approximately 1 ml of blood was transferred into a PCR sample tube and immediately stored at −20°C. The RDT and first blood slide reading were recorded immediately. A second slide reading and PCR test were conducted within a week of the initial blood draw. Results based on the RDT and first blood slide reading were provided to the patient within an hour of sampling and if found infected, malaria treatment provided (artemisinin-based combination drugs and primaquine as appropriate to specific infection).

The study sampling continued in a consecutive manner until the target sample size was obtained. The minimum sample size (n = 400) for accurately estimating sensitivity and specificity [18] was based on the reported lowest RDT sensitivity value (84.4% for *P. vivax*), the Mimika 2012 estimated malaria incidence of 0.53 infections per person-year in the resident population, and an absolute precision value of 0.05. Those cases with incomplete information regarding symptom presentations or laboratory findings, and infections with only *P. ovale* or *P. malariae* parasites based on qPCR were excluded from the final analysis.

### Rapid diagnostic test

The XW-P07 is a rapid, qualitative immunoassay, lateral flow cassette device that uses 5 μl whole blood for the detection of *P. falciparum*-specific histidine rich protein-2 (*P. falciparum*-HRP2), *P. vivax*-specific *Plasmodium* lactate dehydrogenase (*P. vivax*-pLDH), and pan-specific pLDH for all *Plasmodium* species (i.e., *P. malariae* and *P. ovale*).

**Figure 1:**
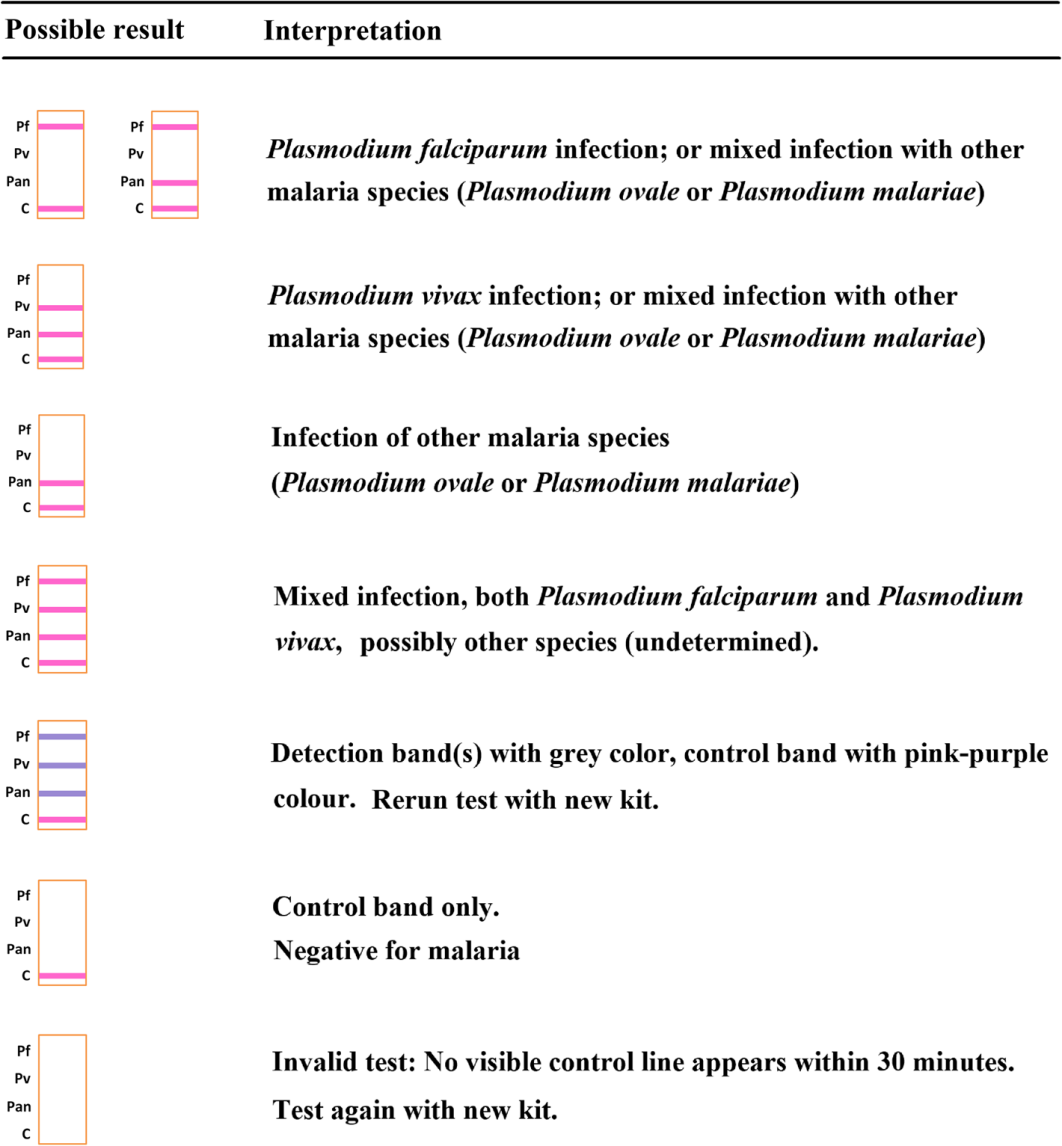
**XW-P07 test criteria for determination of malaria infection.**

The RDT (Batch 91155A, expiration 31 July 2015) was used according to the manufacturer’s instructions provided in the product insert. Reading and interpretation of test results were done by two trained technicians and interpreted by consensus within the specified 15–30 min test window. No test was scored beyond the 30 min limit. Any test that failed to produce a control band was considered invalid and the test repeated. Test interpretation criteria are provided in Figure 1. As example, if matched microscopy or qPCR detected only *P. vivax*, while the RDT showed all four bands reactive, the interpretive result of the RDT would be *P. falciparum* false positive, *P. vivax* true positive, *P. ovale* and/or *P. malariae* as equivocal and undetermined. Given the low prevalence of the latter two species, this study restricted performance analysis on *P. falciparum* and *P. vivax* infections only.

### Microscopy

Microscopy and qPCR were used as the reference standards in this study. With each RDT, a matching thick and thin blood film (one slide per sample) was prepared and stained with Giemsa solution (1:10 dilution for approximately 20 min). Slides were examined using a compound light microscope under x1,000 oil-immersion magnification by a qualified laboratory technician in the clinic. All blood films were examined for a minimum of 100 high-magnification fields before being recorded as either negative for malaria parasites and for the detection of low density mixed species infections. The parasite densities were estimated for each sample counted separately by parasite species. Parasite numbers were reported per 200 white blood cells (WBC) to estimate parasite density per μl of blood, assuming a standard mean WBC count of 8,000/μl blood. Samples were also categorized into one of four groups based on overall parasite density (1–100; 101–1,000; 1,001-10,000; and 10,001-100,000 parasites/μl blood).

All slides were subsequently examined by an independent expert malaria microscopist as an observer-blinded cross-check and confirmation of first examination. A third, observer-blinded expert microscopist was used for slides where there was either discordant or significant discrepancies in findings (infection density or parasite species discrepancies) between the first and second examiners. In such cases, the results for the third examination were regarded as final. All microscopists in this study were experienced in malaria slide preparation and diagnosis, and certified through quality assurance procedures.

### Quantitative real-time PCR

Quantitative real-time PCR (qPCR) amplification was performed using a Rotor-Gene Q (Qiagen, Hilden, Germany). Parasite DNA (if present) was extracted from whole blood using Qiagen DNeasy Blood and Tissue Kit (Qiagen, Hilden, Germany), according to the manufacturer’s instructions. Extracted DNA was eluted in a final volume of 100 μl for the first elution and 50 μl for the second. DNA concentration and purity were measured using a nanophotometer (Implen GmBH, Munich, Germany) and then immediately stored at −20°C. Amplification was carried out in a 20 μl reaction volume, containing 10 μl SYBR Green (BioRad, California, USA), 0.3 mM of each primer, 30 ng DNA template, and nuclease-free water (Promega, Wisconsin, USA). A pair of primers was used to amplify the 18S rRNA gene sequences: PL1473F18 (5′TA CGA ACG AGA TCT TAA-3′) and PL1679R18 (5′GTT CCT CTA AGA AGC TTT-3′) for the four *Plasmodium* species.

The conditions for the qPCR consisted of initial denaturation at 95°C for 10 minutes, 40 cycles amplification at 95°C for 10 seconds, 50°C for 5 seconds, and 72°C for 20 seconds each, with fluorescence acquisition at the end of each extension step. The melt program consisted of 2 minutes at 95°C and 68°C each, followed by a stepwise temperature increase of 0.2°C/s until 90°C, with fluorescence acquisition preformed at each temperature transition. *Plasmodium* species differentiation was achieved using melting curve analysis. Above steps and subsequent data interpretation for qPCR followed Mangold *et al*. [19] for detection and identification of each parasite species. A melting curve analysis with the four species controls with no template control was used in the RDT performance evaluation. Graphics were generated using default software program of Rotor-Gene Q Series Software to compare against standard melting temperatures for *P. falciparum* 75.5-77.5°C, *P. vivax* 79.0-81.0°C, *P. ovale* 77.5-79.0°C, and *P. malariae* 73.5-75.5°C [19].

### Statistical analysis

Data was analyzed using Stata 12.0 software (Stata Corporation, College Station, Texas, USA, license no. 08762859510). RDT performance was calculated compared with matched microscopy and qPCR results with 95% confidence intervals (CI) for the following values: sensitivity (Sn), specificity (Sp), positive predictive value (PPV), negative predictive value (NPV), positive likelihood ratio (PLR), negative likelihood ratio (NLR), and Kappa score. RDT sensitivity was also calculated based on parasite density. Odds ratios and confidence intervals were calculated for correlation between low parasite counts (≤100/μl blood) and RDT false negative results based on matching microscopy and qPCR results; as well as between low parasite counts and body temperature at the time of blood sampling. For statistical inference, the Fisher’s exact test was used for interpretation of false negative RDT results between malaria infections with densities above and below 100 parasites/μl blood with significance set at *p*-value of <0.05.

### Ethical review

All observations and reporting contained herein are based on informed consent by volunteers (adults or minors with their legal guardian’s consent) prior to physical and laboratory examinations. A unique medical record number was used as an identifier of each sample and all information was kept confidential throughout the study. Ethical review and clearance was obtained from the Medical and Health Research Ethics Committee (Reference no. KE/FK/320/EC), Faculty of Medicine, Gadjah Mada University, Yogyakarta, Indonesia.

## Results

**Figure 2:**
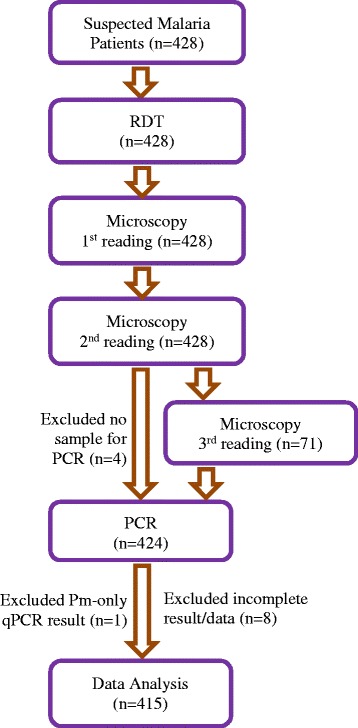
**Distribution of blood samples for determining XW-P07 performance.**

Between 10 April and 14 May 2014, 428 suspected malaria patients attending a local clinic in Kuala Kencana, Mimika, voluntarily provided blood samples (Figure 2). Of these initial samples, eight had incomplete data, one case had only *P. malariae* infection, and four lacked matching qPCR testing; therefore, only 415 samples were included in the study and analysis. No adverse events were recorded with any patient during the blood draw process or thereafter. In this study, 80% (n = 331) of patients reported ‘fever’, while only 47.5% (n = 197) actually had a body temperature ≥37.5°C at the time of blood sampling. Baseline characteristics of study population are shown in Tables 1 and 2.

**Table 1:** **Baseline characteristics of analyzed samples based on microscopy diagnosis**

**Male**	**302/415 (72.8%)**
Mean age in years (range)	28.9 years (6 months–76 years)
Temperature ≥37.5°C or ‘fever’	197/398 (49.5%)
Mean *Plasmodium falciparum* parasite/μl blood (range)	11,388 (40–68,040)
1-100/μl	5/88 (5.7%)
101-1,000/μl	18/88 (20.5%)
1,001-10,000/μl	34/88 (38.6%)
10,001-100,000/μl	31/88 (35.2%)
Mean *Plasmodium vivax* parasite/μl blood (range)	3,143 (40–27,000)
1-100/μl	14/85 (16.5%)
101-1,000/μl	25/85 (29.4%)
1,001-10,000/μl	42/85 (49.4%)
10,001-100,000/μl	4/85 (4.7%)

Positive samples were divided between *P. falciparum* and *P. vivax* infections, and those with mixed species infections were counted separately as *P. falciparum* and *P. vivax*.

**Table 2:** **Signs and symptoms of suspected malaria cases with and without parasitaemia based on microscopic diagnosis**

**Signs and symptoms**	**Parasitaemia**	**Parasitaemia**
	**Present (n = 168)**	**Absent (n = 247)**
Fever	154 (91.7%)	177 (71.7%)
Chills	68 (40.5%)	52 (21.0%)
Sweating	13 (7.7%)	12 (4.9%)
Nausea	71 (42.3%)	60 (24.3%)
Vomiting	35 (20.8%)	32 (13.0%)
Diarrhea	24 (14.3%)	41 (16.6%)
Headache	71 (42.3%)	57 (23.1%)
Myalgia	52 (31.0%)	55 (22.3%)
Paroxysm: combination chills, fever, & sweating	12 (7.1%)	5 (2.0%)
≥3 outcomes	97 (57.7%)	77 (31.2%)
≥4 outcomes	60 (35.7%)	26 (10.5%)
Temp ≥37.5°C	112 (66.7%)	85 (34.4%)

The RDT cassettes used in this study showed clear line intensities and had good background clearing after addition of the buffer solution; however, 3 out 415 tests failed to produce a ‘control’ line and thus had to be repeated with a new cassette using the same blood sample. Laboratory test findings are shown in Table 3. RDT results found 21.0% (87/415) of samples reactive for *P. falciparum*, 14.5% (60/415) for *P. vivax*, and 1.2% (5/415) with mixed *P. falciparum*/*P. vivax* infections. Microscopy produced a total SPR of 40.5% (168/415); with *P. falciparum* slightly more common than *P. vivax*. To determine RDT performance using matching microscopy, positive slides were divided between *P. falciparum* and *P. vivax*; and those with mixed species infections were counted twice, separately as *P. falciparum* and *P. vivax*. Composite determinations from first, second, and third slide (if required) examinations were used to calculate final RDT performance. A total of 64 blood slides (15.4%) out of 415 observations required a third reading for arbitration over any discrepant results from the initial first and second examinations. When compared with matched qPCR, the microscopy error rates for the first and second examinations were 12% and 8%, respectively; therefore, the parasite density determinations from the second reading were used for the final analysis. Overall, compared to the RDT, microscopy resulted in an increased detection of infection, with Kappa scores (inter-procedure agreement) between composite microscopy and qPCR of 0.97 for *P. falciparum* and 0.92 for *P. vivax*.

**Table 3:** **Summary RDT results compared with matching microscopy and qPCR in 3×3 table**

**RDT**	**Microscopy Pf**			**Microscopy Pf, Pv**	**Microscopy Pf, Pm**	**Microscopy Pv**			**Microscopy NP**		
**Result**	**PCR**	**PCR**	**PCR**	**PCR**	**PCR**	**PCR**	**PCR**	**PCR**	**PCR**	**PCR**	**PCR**
	**Pf**	**Pf, Pv**	**NP**	**Pf, Pv**	**Pf, Pm**	**Pv**	**Pv, Pm**	**NP**	**Pf**	**Pv**	**NP**
Pf	32	0	0	1	0	0	0	0	0	0	8
Pf, Pan	40	2	0	2	2	0	0	0	0	0	0
Pv	1	0	0	0	0	14	0	0	0	0	2
Pv, Pan	0	0	0	0	0	43	0	0	0	0	0
Pan	1	0	0	0	0	0	0	0	0	0	0
Pf, Pv, Pan	0	0	0	2	0	3	0	0	0	0	0
No parasites	3	0	2	0	0	18	1	1	2	8	227
Σobservations	77	2	2	5	2	78	1	1	2	8	237

NP = No parasites. Pf: *Plasmodium falciparum*, Pv: *Plasmodium vivax,* Pm: *Plasmodium malariae.*

Table 4 provides final RDT performance measures and 95% CI compared with microscopy for detecting *P. falciparum* was Sn 92%, Sp 96.6%, PPV 88%, NPV 97.8%, with Kappa score 0.87; and for *P. vivax*: Sn 72.9%, Sp 99.1%, PPV 95.4%, NPV 93.4%, with Kappa score 0.79. Final RDT performance compared with qPCR for detecting *P. falciparum* was Sn 92%, Sp 96.6%, PPV 88%, NPV 97.8%, with Kappa score 0.87; and for *P. vivax*: Sn 66%, Sp 99.1%, PPV 95.4%, NPV 90.9%, with Kappa score 0.73.

**Table 4:** **RDT performance compared with matching microscopy and qPCR (n = 415) for all infections regardless of parasite density**

**RDT performance**	***Plasmodium falciparum*** **(n = 88 microscopy +)**	***Plasmodium vivax*** **(n = 85 microscopy +)**	***Plasmodium falciparum*** **(n = 88 qPCR +)**	***Plasmodium vivax*** **(n = 94 qPCR +)**
Prevalence	21.2%	20.5%	21.2%	22.6%
Sn*	92% (84.3-96.7%)	72.9% (62.2-82%)	92% (84.3-96.7%)	66% (55.5-75.4%)
Sp*	96.6% (94.1-98.3%)	99.1% (97.4-99.8%)	96.6% (94.1-98.3%)	99.1% (97.3-99.8%)
PPV*	88% (79.6-93.9%)	95.4% (87.1-99%)	88% (79.6-93.9%)	95.4% (87.1-99.0%)
NPV*	97.8% (95.6-99.1%)	93.4% (90.3-95.8%)	97.8% (95.6-99.1%)	90.9% (87.3-93.7%)
PLR*	27.4 (15.3-49.1)	80.2 (25.8-249)	27.4 (15.3-49.1)	70.6 (22.7-220)
NLR*	0.08 (0.04-0.17)	0.27 (0.19-0.39)	0.08 (0.04-0.17)	0.34 (0.26-0.46)
Kappa	0.87	0.79	0.87	0.73

Sn = sensitivity; Sp = specificity; PPV = positive predictive value; NPV = negative predictive value; PLR = positive likelihood ratio; NLR = negative likelihood ratio.

*95% CI.

RDT sensitivity for detecting *P. falciparum* or *P. vivax* was different depending on parasite density as measured from peripheral blood (Table 5). This difference was statistically significant for *P. falciparum* (*p* = 0.02) and *P. vivax* (*p* <0.001). The RDT achieved 100% sensitivity at high parasite densities ≥4,800 parasites/μl blood for *P. falciparum* and at a lower threshold of ≥640 parasites/μl for *P. vivax*. Parasite densities at or below 100/μl increased the probability of the RDT producing a false negative finding; thus, impacting the overall malaria detection performance of the test. When compared with microscopy, the odds ratio with low parasite densities resulting in RDT false negative findings compared to densities >100/μl was 10.4 (*p* = 0.05) for *P. falciparum*, and 18 (*p* <0.001) for *P. vivax*. Similarly, when compared with qPCR, the odds ratio for false negative results at low parasite densities was 6.5 (*p* = 0.2) for *P. falciparum* and 16.4 (*p* <0.001) for *P. vivax* (Table 6). In RDT comparisons, significant differences were seen with *P. vivax* compared with microscopy and qPCR, but only borderline significance with *P. falciparum* and microscopy. However, after excluding *P. vivax* densities of ≤100/μl, the RDT performance sensitivity compared with qPCR increased from 66% (95% CI 55.5-75.4%) to 72.8% (61.8-82.1%), along with adjusted Sp 99.1% (97.3-99.8%), PPV 95.2% (86.5-99%), NPV 93.5% (90.3-95.9%), PLR 77.7 (25–242), NLR 0.3 (0.2-0.4), and Kappa score 0.79. Similarly, when removing the lower density *P. falciparum* infections, the RDT performance compared with qPCR remained nearly the same, increasing sensitivity only slightly from 92% (84.3-96.7%) to 92.9% (85.1-97.3%), followed by Sp 96.6% (94–98.3%), PPV 87.6% (79–93.7%), NPV 98.1% (96–99.3%), PLR 27.5 (15.3-49.3), NLR 0.07 (0.03-0.16), and Kappa score 0.88.

**Table 5:** **RDT percent test sensitivity by parasite density based on microscopy for** ***Plasmodium falciparum*** **and** ***Plasmodium vivax***

**Parasite density in blood**	***Plasmodium falciparum***		***Plasmodium vivax***	
	**n = RDT+/microscopy+**	**Sn (%)**	**n = RDT+/microscopy+**	**Sn (%)**
1-100/μl	3/5	60	3/14	21.4
101-1,000/μl	14/18	77.8	13/25	52
1,001-10,000/μl	33/34	97	42/42	100
10,001-100,000/μl	31/31	100	4/4	100
Cut-off for 100% Sensitivity	4,800 parasites/μl		640 parasites/μl	

Sn = sensitivity.

**Table 6:** **Odds ratios of RDT false negative results comparing parasite densities below and above 100/μl blood for** ***Plasmodium falciparum*** **and** ***Plasmodium vivax***

**Microscopy**	**False**	**True**	**Odds ratio (95% CI)**	***p*** **-value**
	**Negative**	**Positive**		
Pf ≤100/μl	2	3	10.4 (1.4-77.2)	0.05
Pf >100/μl	5	78		
Pv ≤100/μl	11	3	18.0 (4.4-74.5)	<0.001
Pv >100/μl	12	59		
**qPCR**	**False**	**True**	**Odds ratio (95% CI)**	***p*** **-value**
	**Negative**	**Positive**		
Pf ≤100/μl	1	3	6.5 (0.5-77.3)	0.2
Pf >100/μl	4	78		
Pv ≤100/μl	10	3	16.4 (3.9-68.6)	<0.001
Pv >100/μl	12	59		

Pf: *Plasmodium falciparum*, Pv: *Plasmodium vivax.*

Normal body temperature recorded at the time of blood sampling was significantly associated with lower parasite density in both *P. falciparum* and *P. vivax* infections. The odds ratio of having a normal body temperature (<37.5°C) and lower parasite densities (≤100/μl) was 12 (95% CI: 1.3-113.7; *p* = 0.02) for *P. falciparum*, and 5.5 (95% CI: 1.6-18.9; *p* = 0.004) for *P. vivax*.

## Discussion

Definitive diagnosis and confirmation of disease status is the cornerstone of evidence-based medicine. Many malaria-endemic areas of the world lack sufficient capacity and resources to accurately diagnose the infection and where reliance on the presentation of clinical signs and symptoms alone are inadequate and imprecise indicators of specific disease. The WHO recommendations for procurement of malaria RDTs are currently based on the attainment of a set of minimum performance criteria (e.g., detection rate/panel detection score, specificity, invalid rate, etc.) in the WHO Malaria RDT Product Testing Program [16] and recommendations established by the WHO Malaria Policy Advisory Committee in 2012 [20]. Products that fail to meet the full set of minimum performance criteria are not eligible for procurement by WHO. To some degree, many other organizations and government procurement authorities also follow the WHO guidelines for product selection. Based on published findings [10,16], the XW-P07 RDT has not been tested by the standardized, laboratory-based WHO program; therefore, an evaluation regarding its performance for detection of malaria compared to microscopy and qPCR in a point-of-care operational setting was deemed prudent and essential.

The RDT cassettes were easy to use and provided distinct, easy to interpret test lines. In only 3 tests did the RDT fail to show a control line, producing an ‘invalid rate’ of 0.72%, well within the acceptable limit (<5%) established by WHO [16]. The RDT showed sensitivity and specificity values of >90%, PLR >10, NLR <0.1, and Kappa >0.8 for detecting *P. falciparum* infections when compared with microscopy and qPCR. On the other hand, for *P. vivax*, the RDT showed the same specificity >90% and PLR >10; while the overall sensitivity was much lower when compared with microscopy and qPCR (73% and 66% respectively), with NLR >0.1 and Kappa score slightly <0.8. The test specificity for both parasite species easily met the WHO recommended minimum performance criteria of >90% (i.e., less than 10% false positive rate) detection at 200 parasite/μl [20].

An unpublished product evaluation by the XW-P07 manufacturer reported findings from 251 samples compared to microscopy (SPR 16.3%) showing Sn and Sp of 100% for both *P. falciparum* and *P. vivax* [8]. A study performed in Lampung Province, Sumatra, with 400 samples (SPR 36%) showed Sn 91% (85-97%), Sp 99% (98-100%), PPV 98% (95-100%), NPV 97% (95-99%) for *P. falciparum*; and Sn 84% (75-92%), Sp 100%, PPV 100%, NPV 96% (94-98%) for *P. vivax* based on comparisons with matched microscopy [9]. The study in Mimika demonstrated different predictive values for both parasites compared to previous investigations which may have been influenced by the different disease prevalence in each study [21]. Any direct comparison between studies on RDT performance may be compromised by other factors related to location, sample population, background malaria exposure, and degree of acquired partial immunity in the target populations. This study showed that RDT sensitivity is clearly influenced by parasite density, not an unexpected finding based on testing of other products [10,16]. RDTs have been shown to produce lower sensitivity in areas with more frequent low parasite densities [22,23]. In this study, body temperature was significantly associated with parasite density as measured in peripheral blood – normal temperatures at time of exam produce lower infection densities while corroborating other observations that a rise in body temperature is correlated with an increase in parasite density [24,25].

This product evaluation was not without some underlying limitations. Firstly, this study only used RDT cassettes from a single lot number; thus, possible inter-lot variability in performance between product production periods was not assessed. This study was conducted in a reasonably controlled setting with trained laboratory technicians; therefore, extrapolation of findings to areas under more demanding environmental conditions and clinical expertise (e.g., remote primary health care clinics) should be made with caution. Albeit relatively uncommon infections, *P. ovale* and *P. malariae* were not specifically included in the panel assay (only as a pan-specific pLDH for all *Plasmodium* spp.); however, the majority of these infections in Papua are often coincident (mixed) with other plasmodial species. In this study, three *P. malariae* infections were either mixed with *P. falciparum* (two cases) or *P. vivax* (one case). Lastly, a set minimum of 100 high magnification fields were used for blood examination of films which possibly contributed to the relatively high (15.4%) discordant results between the first 2 microscopists. The detection accuracy would have likely been enhanced had each reader examined a minimum of 200 fields.

Various host and parasite factors are possible reasons for varying RDT performance values between different malaria endemic populations [10,26-31]. Greater sensitivity is a desired attribute and maybe more important compared to test specificity to ensure malaria infections are correctly diagnosed and promptly treated to avoid development of disease complications and more severe infections when left untreated. Undetected cases due to false negative results also contribute a continuing source of gametocyte carriers (reservoirs) for sustaining malaria transmission in an area [31]. Conversely, higher test sensitivity may result in lower test specificity (higher false positive results), thereby increasing unnecessary malaria treatments [32]. Typically, *P. falciparum* infections have been regarded as the only human plasmodial species responsible for common causes of severe morbidity and mortality; however, that general perception has changed and been challenged by a number of recent studies showing that *P. vivax* can produce substantially greater morbidity manifesting as severe infections, causing acute and chronic anaemia, and ultimately resulting in death [33-36]. It is because of these heightened concerns regards the higher likelihood of more severe outcomes caused by *P. vivax* infections, that accurate diagnosis of this species becomes an even greater priority to ensure early and effective treatment. An RDT that lacks the necessary sensitivity for detecting *P. vivax* (>90% preferred) and poor exclusion power (a negative diagnostic likelihood ratio of >0.1) to adequately rule out infection presents a distinct disadvantage to both patient and health care provider in areas where the parasite is common.

This study showed that XW-P07 has a significantly lower detection rate for *P. vivax* than for *P. falciparum*, even when excluding low density infections below 100 parasites per μl/peripheral blood. Published WHO product evaluation on different malaria RDT products submitted for testing has shown that targeting *P. falciparum* HRP2 has the highest and most consistent detection rate [16]. However, this conflicts with other findings in which HRP2-based RDTs have shown a lower performance value than products using pLDH for detecting *P. falciparum*, as the pLDH capture system is not affected by a possible ‘prozone’ effect, parasite antigen polymorphisms or gene deletions [37-41]. In Myanmar, a study comparing a commonly used RDT utilizing HRP2 and pan pLDH compared with microscopy demonstrated *P. vivax* and *P. malariae* were detected to a far lesser extent (lower sensitivity, NPV, and NLR) than *P. falciparum* [42]. For *P. vivax* detection, separate aldolase and pLDH targeting RDTs have been shown to perform differently depending on the samples tested, increasing the risk of misdiagnosis and therefore suggesting that test sensitivity for *P. vivax* can be improved by using a combination of both aldolase and pLDH in a single RDT [43]. The RDT evaluated in this study only utilizes pLDH for detection of *P. vivax*, thus one possible explanation for the inferior sensitivity seen. The XW-P07 showed considerably lower sensitivity and a poor negative likelihood ratio, both measures falling below general acceptance criteria for detecting *P. vivax* infections. This is especially problematic for infections presenting with lower parasitaemia; thus, RDT results with such limitations must be interpreted with caution if the test is the sole method of diagnosis, particularly given the importance and high prevalence of vivax malaria in the Mimika area.

Furthermore, as a four-band RDT with one control line and three test lines (Figure 1), the XW-P07 relies on more antigen/antibody reactions using a single buffer compared to other RDTs with only three indicator bands. In areas where the prevalence of *P. ovale* and *P. malariae* is relatively low, a three-band RDT with better overall performance and able to differentiate *P. falciparum* and *P.vivax*, or possibly a *P. falciparum*/Pan-malaria test, may be a better format. Whenever possible, it would also be prudent and strongly advised to back-up all RDT diagnosis, regardless of RDT performance rating, with matched blood films and proficient microscopic examination.

A recent study in Flores, Indonesia, found that qPCR revealed almost eight times more *Plasmodium* infections when compared with microscopy, taking into account the high number of sub-microscopic infections in a relatively low transmission area [44]. Molecular methods are universally accepted as more sensitive than microscopy alone. However, PCR (e.g., multiplex, real-time or conventional) requires a sophisticated laboratory setting, trained technicians, entails a longer diagnosis time and higher costs to support the system; thereby precluding its routine use in most malaria endemic areas of the world - Indonesia and Mimika included. New or improved diagnostic methods are in development [45-48] that may vastly improve diagnostic capabilities and accuracy in challenging locations and basic health care settings. However, until superior, easy-to-use alternatives are available, both the RDT and microscopy, alone or in combination, will remain the mainstays for routine point-of-care malaria diagnosis.

In areas with high prevalence of *P. vivax* infection, from a cost-effectiveness point of view, standard expert microscopy should continue to be used as the reference gold standard for malaria diagnosis despite the likelihood of missing some low density parasitemia and sub-microscopic infections. With skilled technicians and experienced health care providers, microscopy has more than sufficient, if not excellent, diagnostic capacity in most instances. All public-funded health facilities and private clinics in the Mimika Regency must either begin, or ensure the continuation of microscopy, as their primary means of malaria diagnosis. Microscopy should be used for routine confirmation of all RDTs performed in clinical settings whenever possible. Lastly, in many circumstances without external funding to support procurement and routine access to RDTs, the sustainability for maintaining these devices in all clinics is vulnerable to supply disruptions without adequate safeguards and reliable logistical support in place. The use of microscopy, even absent the aid of electricity in the most remote areas, is a sustainable approach and within the supportive framework of the Indonesian health care system. Moreover, providing basic electrical power using efficient solar capture devices and battery storage for a microscope and basic clinical equipment is well within the means of most local health budgets. The availability and routine use of microscopy also enables a facility to diagnose other important endemic diseases (e.g., tuberculosis, lymphatic filariasis, intestinal helminths and protozoa) and hematological conditions and indicators without the need of more sophisticated and costly techniques and medical instrumentation.

As malaria represents one of the leading and arguably most important health concern in the Mimika area, various health program stakeholders should continue or adopt a policy of investment in the procurement and maintenance of quality microscopes, the recruitment of additional trained laboratory technicians, and organize regular refresher training on microscopy and RDT proficiency. Nevertheless, for logistical and operational rationale, the use of RDTs will continue to play a valuable and important role in remote areas for first line diagnosis of malaria. Health care facilities in remote locations with limited laboratory capacity should continue to use high quality malaria RDTs combined with evidence from good clinical observations until microscopy can be introduced.

## Conclusions

As a point-of-care device, the XW-P07 provided good test sensitivity, specificity, positive and negative predictive values and likelihood ratios, as well as inter-procedure agreement for detecting *P. falciparum* infections. For *P. vivax* infections, the test provided acceptable specificity and positive likelihood ratio, but with lower sensitivity, negative likelihood ratio (below test acceptance criteria), and inter-procedure agreement comparison. Low parasite blood densities (≤100 parasites/μl), especially with *P. vivax*, increased the probability of false negative test results. Normal body temperature was strongly associated with the incident of lower parasitaemia, further complicating diagnosis. The RDT meets WHO minimum performance criteria for test specificity (>90%) and invalid rate (<5%). As vivax malaria is a very common and important parasitic infection in the population studied, all primary health centers in the Mimika Regency should begin, or continue using, expert-level and quality assured standard microscopy in a sustainable manner for greater accuracy in malaria detection.
